# *Neisseria gonorrhoeae* in Six University Hospital STI Clinics in Sweden – Distribution, Antimicrobial Resistance and Treatment

**DOI:** 10.2340/actadv.v106.adv-2025-0250

**Published:** 2026-06-24

**Authors:** Sinja Kristiansen, Annika Johnsson, Christian Steczkó Nilsson, Elisabet Nylander, Karin Sanner, Miryam Spångerud, Petra Tunbäck, Arne Wikström

**Affiliations:** 1 Department of Dermatology and Venereology, Skane University Hospital, Lund University, Malmö, Sweden; 2 Department of Dermatology and Venereology, Uppsala University Hospital, Uppsala University, Uppsala, Sweden; 3 Department of Public Health and Clinical medicine, Umeå University, Umeå, Sweden; 4 Department of Dermatology and Venereology, Linköping University Hospital, Linköping, Sweden; 5 Department of Dermatology and Venereology, Institute of Clinical Sciences, Sahlgrenska Academy, University of Gothenburg, Gothenburg, Sweden; 6 Department of Dermatology and Venereology, Sahlgrenska University Hospital, Gothenburg, Region Västra Götaland, Sweden; 7 Department of Dermatology and venereology, Karolinska University Hospital and Department of Medicine, Karolinska Institute, Solna, Sweden

**Keywords:** *Neisseria gonorrhoeae*, antimicrobial resistance, sexually transmitted infection, STI, Nucleic acid amplification test, NAAT

## Abstract

Gonorrhoea is a sexually transmitted infection caused by *Neisseria gonorrhoeae* with an increasing incidence and a widespread antimicrobial resistance. The aim was to investigate the case burden, distribution of infection site, management and antibiotic resistance in *N. gonorrhoeae* cases in 6 university hospitals in Sweden. Diagnosis of *N. gonorrhoeae* was made by nucleic acid amplification tests (NAAT). Included were 1,060 cases with 27.3% women, 72.4% men and 4 transgender persons, constituting around one third of all 3,356 *N*. *gonorrhoeae* cases in Sweden 2022. The NAAT test for *N. gonorrhoeae* from urine was positive in 51.7% (428/828), 40.4% (428/1,060) among the total included population, from vagina/cervix in 87.9% (246/280), 23.2% (246/1,060) in the total population and from rectum in 66.1% (353/534), 33.3% (353/1,060) among all included cases, with 78.9% (277/351) positive rectal samples in men and 21.1% (74/351) in women (*p*<0.001), the majority being among men having sex with men (76.5%). Antimicrobial resistance was most common against ciprofloxacin, seen in 43.7% (463/1,060) and second most common against azithromycin, in 9.4% (150/1,060). The study elucidates the complexity of handling gonococcal infections regarding relevant test methods, infections in several locations and the problem with antibiotic resistance.

**SIGNIFICANCE**

Gonorrhoea is a sexually transmitted infection with an increasing incidence and widespread antimicrobial resistance. The aim of this study was to investigate the case burden, distribution of infection site, management of diagnosed infections, antibiotic resistance and response to treatment of gonorrhoea. Included were 1,060 gonorrhoea cases from 6 university hospitals in Sweden, comprising around one third of the total number of gonorrhoea cases in Sweden during 2022. The study shows the complexity of handling gonococcal infections regarding relevant test methods, infections in several locations and the rising problem for society with bacteria developing antibiotic resistance.

Gonorrhoea, caused by the gram-negative diplococcus *Neisseria gonorrhoeae*, is a sexually transmitted infection (STI) that primarily affects urogenital mucous membranes, anus and pharynx. Gonorrhoea causes symptomatic urethritis in the majority of men, but only about 50% of women are symptomatic, e.g. by cervicitis. *N. gonorrhoeae* can also infect the mucosa of the rectum and pharynx and more rarely the conjunctiva, as well as cause systemic infections (1). The infection usually remains localized at the initial site of transmission, but untreated the disease may cause complications such as pelvic inflammatory disease (PID) and epididymo-orchitis (2). Transmission is mainly by unprotected intercourse. In penile–vaginal intercourse, the risk of transmission is higher from man to woman due to high bacterial content in ejaculate and a greater mucous area in women (3).

In the EU/EEA, the number of gonorrhoea cases increased from 29,434 in 2008 to 117,985 reported cases in 2019, a rise of about 300% (4). As in the rest of Europe, the number of gonorrhoea cases in Sweden has increased since 2010, except for a temporary decline in 2020 and 2021, probably due to the COVID-19 pandemic (5). Most cases are found among men who have sex with men, and most infections are acquired within Sweden (5).

The antimicrobial resistance (AMR) in *N. gonorrhoeae* is widespread. Data from 73 countries showed decreased susceptibility or resistance to ceftriaxone in 21 (31%) of 68 reporting countries and to cefixime by 24 (47%) of 51 reporting countries. Resistance to azithromycin was present in 51 (84%) of 61 reporting countries and to ciprofloxacin in all 70 (100%) reporting countries. The annual proportion of decreased susceptibility or resistance across countries varied between 0–21% to ceftriaxone and 0–22% to cefixime, and resistance to azithromycin was 0–60% and 0–100% to ciprofloxacin (6). A recent study from EU/EEA showed azithromycin-resistant clones stabilizing at a relatively high level (9.4%), which might influence the recommended ceftriaxone-azithromycin therapy in the European treatment guidelines, but the negligible ceftriaxone resistance was encouraging (7).

All regions in Sweden offer patients home-testing kits for genital *N. gonorrhoeae* ordered via the internet. If the test results are positive the patient is referred to an STI clinic. In Sweden, *N. gonorrhoeae* is covered under the Communicable Diseases Act, which includes obligations to report diagnosed cases and to perform contact tracing (Communicable Diseases Act 2004 : 168). Contact tracing aims to identify and treat infected cases to reduce further spreading. This is especially important given that the infection may be asymptomatic (8).

The aim of this study was to investigate the case burden, distribution of infection site, management of diagnosed infections, antibiotic resistance and response to treatment in *N. gonorrhoeae* cases.

## MATERIALS AND METHODS

Six University Hospitals in the biggest cities of Sweden participated: University Hospital of Umeå, Uppsala University Hospital, Karolinska University Hospital in Stockholm, Linköping University Hospital (including satellite clinics), Sahlgrenska University Hospital in Gothenburg and Skåne University Hospital in Malmö.

All hospitals have laboratories where diagnosis with culture for determining antibiotic resistance are available. In Skåne county, ciprofloxacin resistance determination in NAAT tests was introduced during the study period starting in summer 2022. This is not available as a routine test in any other region in Sweden.

Inclusion criteria were patients ≥18 years testing positive with a nucleic acid amplification test (NAAT) for *N. gonorrhoeae* attending or referred to one of the 6 participating University Hospitals for 1 year, 2022. They were retrospectively included in the study, and their medical records were analysed due to given care for *N. gonorrhoeae*. All participants were given a specific code and analysed on a group level.

Diagnosis of *N. gonorrhoeae* is made by nucleic acid amplification tests (NAAT) from urine in men, vaginal swabs in women and in some cases also by rectal and throat swabs in men and women (1). NAATs offer the highest sensitivity and are used for screening as the primary diagnostic method in Sweden. All included hospitals used validated NAATs, but the methods and branches of NAATs varied. Cultures are thereafter used for positive anatomical locations to determine antibiotic susceptibility, which is important due to resistance in gonococci (1–3, 9).

First-line treatment in the European guidelines (2) is a single dose of 1 g ceftriaxone intramuscularly combined with 2 g oral azithromycin. However, in well-controlled settings with good follow-up and monitoring of resistance, treatment with ceftriaxone alone is recommended (2). In Swedish recommendations, a single dose of 1 g of ceftriaxone alone is used, and patients are recommended a test of cure after around 2 weeks with NAAT and, in symptomatic patients, also a culture (10).

Due to antimicrobial resistance*,* and in order to get complete contact tracing, the national recommendation in Sweden is to refer all *N. gonorrhoeae* cases to an STI clinic.

### Statistics

Statistical analysis was performed in IBM SPSS Statistics Version 30.0.0.0.0. Frequencies of categorical variables was analysed using Pearson χ² test. Fisher exact test was used in cases of small numbers (less than 5 in 1 cell). A 95% confidence interval (CI) and *p*>0.05 was used for associations in significant variables.

The study has been approved by the National Swedish ethical agency, registration code 2020-06659.

## RESULTS

The total number of *N. gonorrhoeae* cases in the 6 university hospitals during 2022 were 1,060 which constituted around one third of all 3,356 *N*. *gonorrhoeae* cases in Sweden during that year (5). Skåne University Hospital contributed 338 cases, Sahlgrenska University Hospital with 282 cases, Karolinska University Hospital with 189 cases, Linköping University Hospital with 113 cases, Uppsala University Hospital with 93 cases and University Hospital of Umeå with 45 cases.

Women with *N. gonorrhoeae* constituted 27.3% (289/1,060), men made up 72.4% (767/1,060) of the *N. gonorrhoeae cases* and 4 cases were declared as transgender persons (Table I). For distribution of gender of sex partners, see Table I.

**Table I.** Background characteristics for the 1,060 included cases of *N. gonorrhoeae* divided by sex and sexual preference based on sex of sex partner when the infection was inherited

	Men*N*, %	Women*N*, %	Trans- gender*N*, %	Total*N*, %	χ^2*^ *p*-value	MSM*N*, %	MSW*N*, %	MST*N*, %	WSM*N*, %	WSW*N*, %	WST*N*, %
Age groups, years											
All	767 (72.4)	289 (27.3)	4 (0.4)	1,060 (100)		500 (47.2)	239 (22.5)	14 (1.3)	276 (26.0)	5 (0.5)	6 (0.6)
18–24	166 (54.1)	138 (45.0)	3 (1.0)	307 (29.0)	<0.001	88 (28.7)	72 (23.5)	3 (1.0)	133 (43.3)	3 (1.0)	2 (0.7)
25–34	334 (79.3)	87 (20.7)	0 (0)	421 (39.7)		221 (52.5)	100 (23.8)	7 (1.7)	82 (19.5)	2 (0.5)	2 (0.5)
35–44	159 (80.3)	39 (19.7)	0 (0)	198 (18.7)		110 (55.6)	43 (21.7)	4 (2.0)	37 (18.7)	0 (0)	2 (1.0)
45–54	65 (81.2)	15 (18.5)	1 (1.2)	81 (7.6)		47 (58.0)	16 (19.8)	0 (0)	14 (17.3)	0 (0)	0 (0)
55–64	28 (75.7)	9 (24.3)	0 (0)	37 (3.5)		21 (56.8)	7 (18.9)	0 (0)	9 (24.3)	0 (0)	0 (0)
>65	12 (100)	0 (0)	0 (0)	12 (1.1)		10 (83.3)	1 (8.3)	0 (0)	0 (0)	0 (0)	0 (0)
Country of acquisition											
Sweden+Nordic countries	333 (67.3)	160 (32.3)	2 (0.4)	495 (46.7)	0.34	187 (37.8)	130 (26.3)	8 (1.6)	155 (31.3)	1 (0.2)	3 (0.6)
Rest of Europe	99 (78.6)	27 (21.4)	0 (0)	126 (11.9)		72 (57.1)	26 (20.6)	1 (0.8)	25 (19.8)	0 (0)	2 (1.6)
Outside Europe	44 (75.9)	14 (24.1)	0 (0)	58 (5.5)		17 (29.3)	22 (37.9)	4 (6.9)	13 (22.4)	1 (1.7)	0 (0)
PrEP	170 (100)	0 (0)	0 (0)	170 (16.1)	<0.001	167 (98.2)	1 (0.6)	1 (0.6)	0 (0)	0 (0)	0 (0)
Known HIV	56 (98.2)	1 (1.8)	0 (0)	57 (5.4)	<0.001	52 (91.2)	2 (3.5)	0 (0)	1 (1.8)	0 (0)	0 (0)
Reason for NG testing											
Symptoms	365 (71.7)	141 (27.7)	3 (0.6)	509 (48.0)	0.009	178 (35.0)	169 (33.2)	8 (1.6)	132 (25.9)	4 (0.8)	4 (0.8)
Check-up due to unprotected sex	233 (78.2)	64 (21.5)	1 (0.3)	298 (28.1)		205 (68.8)	23 (7.7)	4 (1.3)	62 (20.8)	1 (0.3)	1 (0.3)
Contact tracing	165 (66.8)	82 (33.2)	0	247 (23.4)		113 (45.7)	47 (19.0)	2 (0.8)	80 (32.4)	0 (0)	1 (0.4)
Another simultaneous STI	149 (74.9)	48 (24.1)	2 (1.0)	199 (18.8)	0.27	107 (53.8)	38 (19.1)	3 (1.5)	45 (22.6)	1 (0.5)	2 (1.0)
NG within 12 months	104 (91.2)	10 (8.8)	0 (0)	114 (10.8)	<0.001	100 (87.7)	4 (3.5)	0 (0)	10 (8.8)	0 (0)	0 (0)
Another STI (not GC) within 12 months	167 (87.0)	25 (13.0)	0 (0)	192 (18.2)	<0.001	140 (72.9)	24 (12.5)	3 (1.6)	23 (12.0)	0 (0)	2 (1.0)

*Chi-2 test comparing men and women.

MSM:men having sex with men; MST:men having sex with transgender person; MSW:men having sex with women; NG:*Neisseria gonorrhoeae*; PrEP:pre-exposure prophylaxis; STI:sexually transmitted disease; WSM:women having sex with men; WST:women having sex with transgender persons; WSW:women having sex with women.

Male cases infected by a male partner were grouped as men having sex with men (MSM), male cases infected by a female partner were grouped as men having sex with women (MSW), male cases infected by a transgender person were grouped as (MST). Female cases infected by a male partner were grouped as women having sex with men (WSM), female cases infected by a female partner were grouped as women having sex with women (WSW) and female cases infected by a transgender person were grouped as women having sex with a transgender person (WST).

The majority (87.3%) of *N. gonorrhoeae* cases were between 18 and 44 years, see Table I.

Most patients had been infected in the Nordic countries, including Sweden (46.7%). Of the rest, 11.9% had acquired the infection in other European countries and 5.5% had been infected outside Europe, with a similar distribution in the different age groups (Table I).

Of the infected cases 16.1% (170/1,060) used pre-exposure prophylaxis (PrEP) against HIV at diagnosis, all men. Of these, 167 were infected with *N. gonorrhoeae* by a male partner, 1 by a female partner, 1 by a transgender partner, and 1 with a partner of unknown sex. Known positive HIV status was found in 5.4% (57/1,060) of *N. gonorrhoeae* cases, 98.2% (56/1,060) were men and one (1/1,060) was a woman. Of the 56 men, 91.2% were MSM, 3.5% were MSW and 2 cases had partners with unknown sex. One woman was a WSM (Table I).

The reasons for taking a *N. gonorrhoeae* test were symptoms in 48% (509/1,060), wanting a check-up due to unprotected sex in 28.1% (298/1,060) and contact tracing in 23.4% (247/1,060). There was no significant difference between MSW and WSM, but between MSM and WSM (*p*<0.001). It was more common to discover the *N. gonorrhoeae* infection during a check-up for MSM compared to both WSM and MSW (Table I).

Having another STI diagnosed at the same time was seen in 18.8% (199/1,060), with *Chlamydia trachomatis* being the most common, 15.2%. No statistically significant difference was seen between men and women, or with gender of sex partners (Table I).

Diagnosis and treatment for another *gonococcal infection* within the previous 12 months before the actual infection was found in 10.8% (114/1060), and this was more common among men 91.2% (104/114) than women 8.8% (10/114) p<0.001. The majority of previous *N. gonorrhoeae* infections were among MSM 87.7% (100/114), and only 3.5% among MSW and 8.8% among WSM (Table I).

### Positive tests

The NAAT test for *N. gonorrhoeae* from urine was positive in 51.7% (428/828) representing 40.4% (428/1,060) in the total number of included cases. The NAAT test from vagina/cervix was positive in 87.9% (246/280) representing 23.2% (246/1,060) in the cases included (Table II). The NAAT test from rectum was positive in 66.1% (353/534) giving a positivity of 33.3% (353/1,060) among the total number of included cases, in 78.9% (277/351) of men and in 21.1% (74/351) of women (*p*<0.001) with the majority being among MSM (76.5%) NAAT positivity in rectum was also seen in 2 out of 3 transgender persons. The pharynx test was positive in 66.3% (536/809) concluding 50.6% (536/1,060) among the total included number of cases, with no statistical difference between men and women. But when comparing MSM with both WSM and MSW it was more common to have a positive pharynx test among MSM, 55.3%, compared to 29.4% and 12.2% respectively (*p*<0.001). Positivity in the conjunctiva with NAAT test was seen only in 3 cases and analysed only in 15 cases, 20% positivity and only positive in 0.3% (3/1060) among all included cases. Positive NAAT test of the conjunctiva was seen in 2 women and 1 man, where both women also were positive in culture from the conjunctiva, but the man was negative. For further results on positivity in culture, see Table II.

**Table II.** Positive tests for *N. gonorrhoeae* in men, women and transgender persons according to site of infection

	Men*N* (%)	Women*N* (%)	Transgender persons	Analysed*N* (%)	Total*N* (%)	χ^2^ *p*-value
Sex	767 (72.4)	289 (27.3)	4 (0.4)		1,060 (100)	
Nucleic amplification test						
Urine	366 (85.5)	61 (14.3)	1 (0.2)	428/828 (51.7)	428 (40.4)	<0.001
Vagina/cervix	3 (1.2)	242 (98.4)	1 (0.4)	246/280 (87.9)	246 (23.2)	<0.001
Rectum	277 (78.5)	74 (21.0)	2 (0.6)	353/534 (66.1)	353 (33.3)	<0.001
Pharynx	385 (71.8)	149 (27.8)	2 (0.4)	536/809 (66.3)	536 (50.6)	0.24
Conjunctiva	1 (33.3)	2 (66.7)	0 (0)	3/15 (20.0)	3 (1.4)	0.53*
Culture						
Urethra	303 (82.8)	61 (16.7)	2 (0.5)	366/533 (68.7)	366 (34.5)	0.10
Vagina/cervix	0 (0)	134 (99.3)	1 (0.7)	135/248 (54.4)	135 (12.7)	<0.001
Rectum	160 (87.0)	23 (12.5)	1 (0.5)	184/341 (54.0)	184 (17.4)	<0.001
Pharynx	135 (67.8)	63 (31.7)	1 (0.5)	199/595 (33.4)	199 (18.8)	0.51
Conjunctiva	1 (50)	1 (50)	0 (0)	2/7 (28.6)	2 (0.2)	1.00*

The χ^2^ test compares men and women.

*Fisher exact test.

### Resistance

Antimicrobial resistance was most common against ciprofloxacin, seen in 43.7% (463/1060) and second most common against azithromycin, seen in 9.4% (150/1060) (Table III).

**Table III.** Antimicrobial resistance in all *N. gonorrhoeae* cases (*N*=1,056), except the 4 transgender persons

Antimicrobial resistance	Men *N* (%)	Women *N* (%)	Total *N* (%)
Ciprofloxacin resistance Ciprofloxacin sensitivity	372 (80.3) 165 (58.9)	91 (19.7) 115 (41.1)	463 (43.8) 280 (26.5)
Azithromycin resistance Azithromycin sensitivity	127 (84.7) 184 (69.4)	23 (15.3) 81 (30.6)	150 (14.2) 265 (25.1)
Ceftriaxone resistance Ceftriaxone sensitivity	1 (100) 521 (74.5)	0 (0) 178 (25.5)	1 (0.009) 699 (66.2)
Cefixime resistance Cefixime sensitivity	0 (0) 254 (74.9)	0 (0) 85 (25.1)	0 (0) 339 (32.1)

### Treatment

The most common treatment, given in 90.5% (959/1,060), was 1 g of ceftriaxone, followed by 500 mg of ciprofloxacin given in 6.8% (72/1,060) (data not shown).

A test of cure (TOC) was performed with a NAAT in 83.1% (881/1060) and was negative in 78.7% (834/1,060) and positive in 4.4% (47/1060). It was almost as common to leave a NAATs TOC after 7–14 days (29.1%) as after 15–21 days (30.5%) (data not shown). TOC with culture from the positive NAATs test site, was performed in only 2.8% (30/1060), with only one positive test. Most common number of days after the infection for TOC with culture was 7–14 (1.1% (12/1060)), followed by 15–21 (0.9% (10/1,060)).

## DISCUSSION

This study on *N. gonorrhoeae* is to our knowledge the most extensive study in Sweden on case distribution, distribution of infection site, management of diagnosed infections, antibiotic resistance and response to treatment. Data are presented regarding one third of the total number of *N. gonorrhoeae* cases in Sweden during 2022, including cases from all different geographical regions and 6 out of 7 university hospitals, and we therefore believe it is representative.

Men made up the majority of cases infected with *N. gonorrhoeae* with MSM constituting almost 50%. A large systematic review on the prevalence of *N. gonorrhoeae*, with data from all over the world found population-based prevalence data in only 4 countries, making comparisons impossible (11). Data on MSM were identified in 64 studies from 25 countries, but only 26 studies reported both urogenital and rectal samples, with a higher rate of rectal infection in MSM (11). This is in accordance with our study where most rectal infections (76.5%) were seen in MSM, constituting a known risk group for infections with *N. gonorrhoeae* (12, 13). Considering the high occurrence of rectal chlamydia among heterosexual women (14) may indicate an underestimation of rectal gonorrhoeae in females.

The most common reason for testing for *N. gonorrhoeae* was symptoms, in alignment with previous research (15). Nearly 25% were found through contact tracing, mandatory for all *N. gonorrhoeae* infections diagnosed in Sweden. These infections would probably have been undiscovered and could potentially lead to further spread of the infection and long-term complications in the future, in the absence of contact tracing. Sweden is to our knowledge one of few countries in the world with extensive mandatory contact tracing for infections with *N. gonorrhoeae*.

Concomitant infection with another STI was seen in almost 20% of all cases, with *Chlamydia trachomatis* being the most common. This underlines the importance of using dual NAATs for concomitant testing for *Chlamydia trachomatis* and *N. gonorrhoeae*. These tests are now available as commercial tests (16).

A previous infection with *N. gonorrhoeae* was most common among MSM (around 90%), and highest among MSM on PrEP which is in accordance with other studies from Europe (4, 12, 13).

Antimicrobial resistance (AMR) was most common for ciprofloxacin seen in just below 50%. A study from Stockholm showed an increasing resistance in *N. gonorrhoeae* to ciprofloxacin and azithromycin between 2016 and 2022 and they found one case resistant to both cefixime and ceftriaxone (17). This is in alignment with only one case seen in this study resistant to ceftriaxone. This corroborates the results from the large study in Europe investigating AMR in *N. gonorrhoeae* in 2020 and comparing it to 2013 and 2018, where negligible ceftriaxone resistance was seen (7). The treatment recommendation in Sweden for *N. gonorrhoeae* is 1 g ceftriaxone, due to a well-controlled setting with guidelines recommending a TOC. This is different from the European guidelines recommending 1 g ceftriaxone in combination with 2 g of azithromycin, but the guidelines also state that single treatment with ceftriaxone could be an alternative treatment regimen in well-controlled settings (2). TOC was mainly performed with NAAT. Patients in Sweden are routinely booked for a TOC 2 weeks after treatment and are informed that they by the Communicable Diseases Act are not allowed to engage in sexual intercourse before the infection is cleared and they have made a TOC, so the risk of a positive TOC due to reinfection is small.

In conclusion, this paper elucidates the complexity of handling gonococcal infections regarding relevant test methods, infections in several locations, difficulties in assessing test of cure and the problem with antibiotic resistance. The substantial increase in the incidence of these infections reported by most countries really warrants the need for increased, more effective prevention methods and the need for more effective antibiotic alternatives.
